# 3-Chloro­methyl-6,7-dimethyl-1,2-benz­oxazole

**DOI:** 10.1107/S1600536812039700

**Published:** 2012-09-26

**Authors:** M. Kayalvizhi, G. Vasuki, A. Veerareddy, G. Laxminarasimha

**Affiliations:** a Department of Physics, Kunthavai Naachiar Government Arts College (W) (Autonomous), Thanjavur 613 007, India; b Research and Development Laboratories, Suven Life Sciences Limited, Hyderabad 55, Andhra Pradesh, India

## Abstract

In the title compound, C_10_H_10_ClNO, the benzoisoxazole ring is almost planar (r.m.s. deviation = 0.0121 Å) and the chloro substituent in the side chain is anti­clinal relative to the N—C bond of the isoxazole ring. In the crystal, adjacent mol­ecules are linked *via* a pair of weak C—H⋯N hydrogen bonds, forming dimers through a cyclic *R*_2_^2^(8) association.

## Related literature

For the biological and chemical applications of benzoxazoles, see: Ha *et al.* (2010 ▶); Kayalvizhi *et al.* (2011 ▶); Krishnaiah *et al.* (2009 ▶); Qu *et al.* (2008 ▶); Raju *et al.* (2002 ▶); Veerareddy *et al.* (2011 ▶). For graph-set analysis, see: Bernstein *et al.* (1995 ▶).
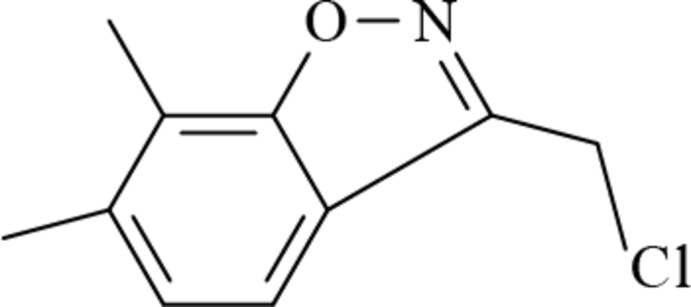


## Experimental

### 

#### Crystal data

C_10_H_10_ClNO
*M* *_r_* = 195.64
Monoclinic, 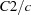

*a* = 20.4938 (15) Å
*b* = 4.1237 (3) Å
*c* = 24.6361 (18) Å
β = 114.151 (3)°
*V* = 1899.8 (2) Å^3^
*Z* = 8
Mo *K*α radiation
μ = 0.36 mm^−1^
*T* = 295 K
0.20 × 0.15 × 0.15 mm

#### Data collection

Bruker Kappa APEXII CCD diffractometer
Absorption correction: multi-scan (*SADABS*; Bruker, 1999 ▶) *T*_min_ = 0.932, *T*_max_ = 0.948
8155 measured reflections
1748 independent reflections
1396 reflections with *I* > 2σ(*I*)
*R*_int_ = 0.035

#### Refinement

*R*[*F* ^2^ > 2σ(*F* ^2^)] = 0.041
*wR*(*F* ^2^) = 0.120
*S* = 1.06
1748 reflections
120 parameters
H-atom parameters constrained
Δρ_max_ = 0.25 e Å^−3^
Δρ_min_ = −0.19 e Å^−3^

### 

Data collection: *APEX2* (Bruker, 2004 ▶); cell refinement: *APEX2* and *SAINT* (Bruker, 2004 ▶); data reduction: *SAINT* and *XPREP* (Bruker, 2004 ▶); program(s) used to solve structure: *SHELXS97* (Sheldrick, 2008 ▶); program(s) used to refine structure: *SHELXL97* (Sheldrick, 2008 ▶); molecular graphics: *ORTEP-3* (Farrugia, 1997 ▶); software used to prepare material for publication: *PLATON* (Spek, 2009 ▶).

## Supplementary Material

Crystal structure: contains datablock(s) I, global. DOI: 10.1107/S1600536812039700/zs2231sup1.cif

Structure factors: contains datablock(s) I. DOI: 10.1107/S1600536812039700/zs2231Isup2.hkl

Supplementary material file. DOI: 10.1107/S1600536812039700/zs2231Isup3.cml

Additional supplementary materials:  crystallographic information; 3D view; checkCIF report

## Figures and Tables

**Table 1:** Hydrogen-bond geometry (Å, °)

*D*—H⋯*A*	*D*—H	H⋯*A*	*D*⋯*A*	*D*—H⋯*A*
C10—H10*B*⋯N2^i^	0.97	2.55	3.479 (3)	160

Symmetry code: (i) 
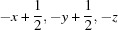
.

**Fig. 1.**
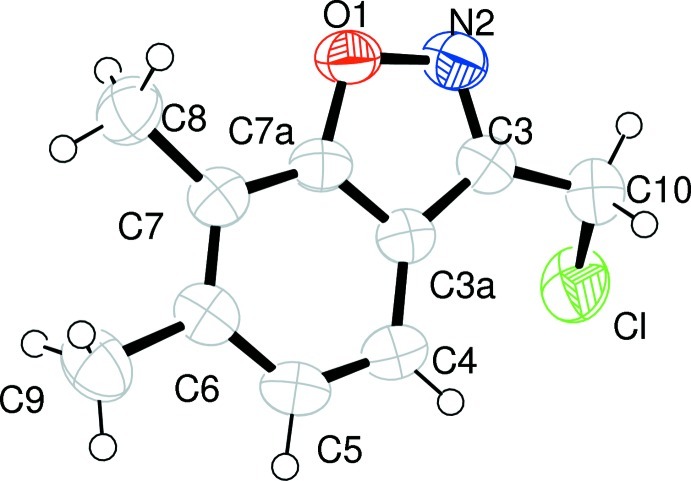
The molecular structure of the title compound showing atom numbering, with displacement ellipsoids drawn at the 50% probability level.

**Fig. 2.**
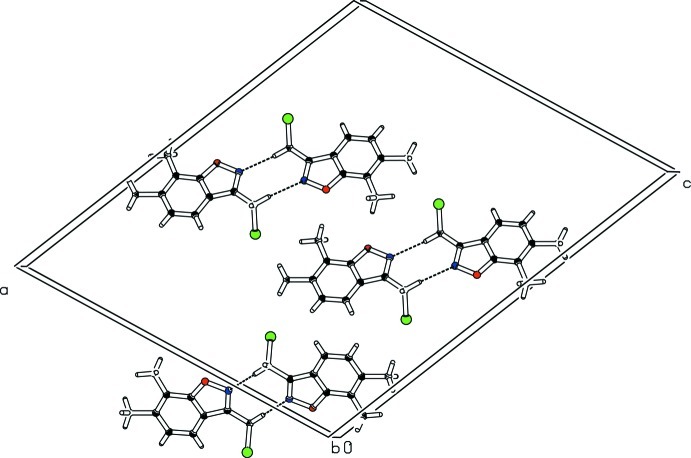
The crystal packing of the title compound in the unit cell, viewed down the b axis, showing the molecular dimers.

## supplementary crystallographic information

### Comment

The benzoxazole ring system is one of the most common heterocycles in medicinal chemistry (Qu *et al.*, 2008). Isoxazole derivatives bearing various substituents are known to have diverse biological activities in pharmaceutical and agricultural areas (Ha *et al.*, 2010). In agriculture applications herbicidal activity has been identified (Raju *et al.*, 2002) as well as fungicidal activities against some plant pathogens (Ha *et al.*, 2010). Some derivatives are also used as semiconductors and as corrosion inhibitors in fuels and lubricants (Raju *et al.*, 2002). They are also important intermediates in the synthesis of many complex natural products (Krishnaiah *et al.*, 2009). Among these compounds, 3-substituted-1,2-benzisoxazole and its derivatives are emerging as potential antipsychotic compounds (Kayalvizhi *et al.*, 2011). Substituted benzoxazoles have been reported to possess diverse chemotherapeutic properties including antibiotic, antimicrobial, antiviral, antitumor and other pharmacological activities (Qu *et al.*, 2008; Krishnaiah *et al.*, 2009). With its extensive uses as a drug for epilepsy, its cost-effective synthesis remained a great challenge for synthetic organic chemists (Veerareddy *et al.*, 2011). In a search for new benzisoxazole compounds with better biological activity, the title compound, C_10_H_10_ClNO, was synthesized and its crystal structure determined, in order to examine the structure–activity effects of the chloromethyl and 6,7-dimethyl substituents on the benzoisoxazole ring.

In the structure of the title compound (Fig. 1) the benzoisoxazole ring is planar with a root mean square deviation of 0.0121 Å. The torsion angle [N2—C3—C10—Cl = 121.31 (19)°] indicates that the side chain is anticlinal looking down the C3—C10 bond. The exocyclic angles C10—C3—C3*a* [129.35 (19)°] and C3—C3*a*—C4 [137.13 (19)°] deviate significantly from the normal values and this may be due to the intramolecular non-bonded interaction between the chlorine atom and an aromatic H atom [Cl···H4 = 3.2582 (8) Å]. In the crystal, adjacent molecules are linked *via* a pair of weak intermolecular C—H···N hydrogen bonds (Table 1) forming dimers through a cyclic *R*^2^_2_(8) association (Bernstein *et al.*, 1995) (Fig. 2).

### Experimental

To a solution of 3,6,7-trimethylbenzo[d]isoxazole-2-oxide (1.0 mol) in methylene dichloride (10 ml) was added POCl_3_ (2.0 mol) dropwise at 20°C over a period of 5 min and stirred for 5 min also at 20°C. Triethylamine (2.0 mol) was then added dropwise at 20°C over a period of 10 min at such a rate that the reaction temperature did not exceed 30°C. The mixture was then stirred at reflux temperature for 48 h and cooled to 10°C. The reaction mixture was washed with chilled water, followed by addition of a 10% Na_2_CO_3_ solution to obtain a neutral pH. The aqueous layer was re-extracted with methylene chloride (2 × 100 ml). The combined organic layer was dried over anhydrous Na_2_SO_4_ and the solvent was removed under vacuum to give the crude product, which was purified by column chromatography and by crystallization (Veerareddy *et al.*, 2011).

### Refinement

All the H atoms were positioned geometrically and treated as riding on their parent atoms, with C—H = 0.93 Å (aromatic), 0.96 Å (methyl) and 0.97 Å (methylene), and refined using a riding model with *U*_iso_(H) = 1.2*U*_eq_ or 1.5*U*_eq_(parent atom).

### Crystal data

**Table:**

C_10_H_10_ClNO	*F*(000) = 816
*M**_r_* = 195.64	*D*_x_ = 1.368 Mg m^−^^3^
Monoclinic, *C*2/*c*	Mo *K*α radiation, λ = 0.71073 Å
Hall symbol: -C 2yc	Cell parameters from 3599 reflections
*a* = 20.4938 (15) Å	θ = 2.2–25.7°
*b* = 4.1237 (3) Å	µ = 0.36 mm^−^^1^
*c* = 24.6361 (18) Å	*T* = 295 K
β = 114.151 (3)°	Block, colourless
*V* = 1899.8 (2) Å^3^	0.20 × 0.15 × 0.15 mm
*Z* = 8	

### Data collection

**Table:**

Bruker Kappa APEXII CCD diffractometer	1748 independent reflections
Radiation source: fine-focus sealed tube	1396 reflections with *I* > 2σ(*I*)
Graphite monochromator	*R*_int_ = 0.035
ω and φ scans	θ_max_ = 25.5°, θ_min_ = 2.2°
Absorption correction: multi-scan (*SADABS*; Bruker, 1999)	*h* = −24→24
*T*_min_ = 0.932, *T*_max_ = 0.948	*k* = −4→4
8155 measured reflections	*l* = −29→29

### Refinement

**Table:**

Refinement on *F*^2^	Primary atom site location: structure-invariant direct methods
Least-squares matrix: full	Secondary atom site location: difference Fourier map
*R*[*F*^2^ > 2σ(*F*^2^)] = 0.041	Hydrogen site location: inferred from neighbouring sites
*wR*(*F*^2^) = 0.120	H-atom parameters constrained
*S* = 1.06	*w* = 1/[σ^2^(*F*_o_^2^) + (0.0575*P*)^2^ + 1.2791*P*] where *P* = (*F*_o_^2^ + 2*F*_c_^2^)/3
1748 reflections	(Δ/σ)_max_ < 0.001
120 parameters	Δρ_max_ = 0.25 e Å^−^^3^
0 restraints	Δρ_min_ = −0.19 e Å^−^^3^

### Special details

**Table:**

Geometry. All esds (except the esd in the dihedral angle between two l.s. planes) are estimated using the full covariance matrix. The cell esds are taken into account individually in the estimation of esds in distances, angles and torsion angles; correlations between esds in cell parameters are only used when they are defined by crystal symmetry. An approximate (isotropic) treatment of cell esds is used for estimating esds involving l.s. planes.
Refinement. Refinement of F^2^ against ALL reflections. The weighted R-factor wR and goodness of fit S are based on F^2^, conventional R-factors R are based on F, with F set to zero for negative F^2^. The threshold expression of F^2^ > 2sigma(F^2^) is used only for calculating R-factors(gt) etc. and is not relevant to the choice of reflections for refinement. R-factors based on F^2^ are statistically about twice as large as those based on F, and R-factors based on ALL data will be even larger.

### Fractional atomic coordinates and isotropic or equivalent isotropic displacement parameters (Å^2^)

**Table:**

	*x*	*y*	*z*	*U*_iso_*/*U*_eq_	
Cl	0.36216 (3)	0.4407 (2)	0.14499 (3)	0.0810 (3)	
O1	0.11696 (8)	0.2766 (4)	0.03641 (6)	0.0579 (4)	
C3A	0.19031 (10)	0.5502 (5)	0.11668 (8)	0.0449 (4)	
C4	0.20916 (11)	0.6941 (5)	0.17240 (9)	0.0535 (5)	
H4	0.2530	0.7972	0.1919	0.064*	
C7	0.07354 (10)	0.3861 (5)	0.11299 (8)	0.0490 (5)	
C7A	0.12460 (10)	0.4049 (5)	0.08966 (8)	0.0464 (5)	
C6	0.09376 (11)	0.5243 (5)	0.16895 (9)	0.0529 (5)	
C3	0.22228 (11)	0.5014 (5)	0.07591 (9)	0.0492 (5)	
C5	0.16064 (12)	0.6770 (5)	0.19698 (9)	0.0560 (5)	
H5	0.1723	0.7708	0.2341	0.067*	
N2	0.18083 (10)	0.3418 (5)	0.02923 (8)	0.0610 (5)	
C8	0.00258 (12)	0.2276 (6)	0.07915 (10)	0.0667 (6)	
H8A	−0.0058	0.0668	0.1038	0.100*	
H8B	0.0026	0.1261	0.0441	0.100*	
H8C	−0.0345	0.3882	0.0679	0.100*	
C10	0.29367 (12)	0.6064 (6)	0.08013 (10)	0.0620 (6)	
H10A	0.2966	0.8413	0.0816	0.074*	
H10B	0.3001	0.5344	0.0452	0.074*	
C9	0.04460 (14)	0.5113 (7)	0.20055 (11)	0.0761 (7)	
H9A	−0.0006	0.6070	0.1759	0.114*	
H9B	0.0656	0.6289	0.2373	0.114*	
H9C	0.0374	0.2895	0.2086	0.114*	

### Atomic displacement parameters (Å^2^)

**Table:**

	*U*^11^	*U*^22^	*U*^33^	*U*^12^	*U*^13^	*U*^23^
Cl	0.0488 (4)	0.1014 (6)	0.0848 (5)	0.0015 (3)	0.0190 (3)	0.0112 (4)
O1	0.0509 (8)	0.0716 (10)	0.0462 (7)	−0.0017 (7)	0.0147 (6)	−0.0086 (7)
C3A	0.0457 (10)	0.0404 (10)	0.0441 (10)	0.0069 (8)	0.0137 (8)	0.0031 (8)
C4	0.0503 (11)	0.0513 (12)	0.0505 (11)	0.0030 (9)	0.0123 (9)	−0.0051 (9)
C7	0.0434 (10)	0.0489 (12)	0.0491 (11)	0.0097 (9)	0.0134 (9)	0.0077 (9)
C7A	0.0471 (10)	0.0449 (11)	0.0403 (9)	0.0087 (8)	0.0107 (8)	0.0016 (8)
C6	0.0528 (12)	0.0534 (12)	0.0510 (11)	0.0142 (9)	0.0196 (9)	0.0066 (9)
C3	0.0498 (11)	0.0469 (11)	0.0497 (11)	0.0075 (9)	0.0191 (9)	0.0045 (9)
C5	0.0624 (13)	0.0571 (13)	0.0445 (10)	0.0093 (10)	0.0178 (10)	−0.0053 (9)
N2	0.0569 (11)	0.0739 (13)	0.0522 (10)	0.0036 (9)	0.0225 (9)	−0.0040 (9)
C8	0.0487 (12)	0.0770 (16)	0.0676 (14)	−0.0030 (11)	0.0170 (10)	0.0022 (12)
C10	0.0605 (13)	0.0608 (14)	0.0678 (14)	0.0006 (11)	0.0295 (11)	0.0058 (11)
C9	0.0754 (16)	0.0931 (19)	0.0708 (15)	0.0116 (14)	0.0411 (13)	0.0039 (13)

### Geometric parameters (Å, º)

**Table:**

Cl—C10	1.776 (2)	C6—C9	1.505 (3)
O1—C7A	1.363 (2)	C3—N2	1.295 (3)
O1—N2	1.417 (2)	C3—C10	1.488 (3)
C3A—C7A	1.372 (3)	C5—H5	0.9300
C3A—C4	1.397 (3)	C8—H8A	0.9600
C3A—C3	1.420 (3)	C8—H8B	0.9600
C4—C5	1.361 (3)	C8—H8C	0.9600
C4—H4	0.9300	C10—H10A	0.9700
C7—C7A	1.387 (3)	C10—H10B	0.9700
C7—C6	1.389 (3)	C9—H9A	0.9600
C7—C8	1.498 (3)	C9—H9B	0.9600
C6—C5	1.406 (3)	C9—H9C	0.9600
			
C7A—O1—N2	107.37 (15)	C6—C5—H5	118.4
C7A—C3A—C4	118.97 (18)	C3—N2—O1	106.82 (16)
C7A—C3A—C3	103.89 (17)	C7—C8—H8A	109.5
C4—C3A—C3	137.13 (19)	C7—C8—H8B	109.5
C5—C4—C3A	117.15 (19)	H8A—C8—H8B	109.5
C5—C4—H4	121.4	C7—C8—H8C	109.5
C3A—C4—H4	121.4	H8A—C8—H8C	109.5
C7A—C7—C6	114.78 (18)	H8B—C8—H8C	109.5
C7A—C7—C8	121.25 (18)	C3—C10—Cl	110.08 (15)
C6—C7—C8	123.97 (19)	C3—C10—H10A	109.6
O1—C7A—C3A	109.88 (17)	Cl—C10—H10A	109.6
O1—C7A—C7	124.63 (18)	C3—C10—H10B	109.6
C3A—C7A—C7	125.48 (18)	Cl—C10—H10B	109.6
C7—C6—C5	120.40 (19)	H10A—C10—H10B	108.2
C7—C6—C9	120.5 (2)	C6—C9—H9A	109.5
C5—C6—C9	119.09 (19)	C6—C9—H9B	109.5
N2—C3—C3A	112.04 (18)	H9A—C9—H9B	109.5
N2—C3—C10	118.61 (19)	C6—C9—H9C	109.5
C3A—C3—C10	129.35 (19)	H9A—C9—H9C	109.5
C4—C5—C6	123.19 (19)	H9B—C9—H9C	109.5
C4—C5—H5	118.4		
			
C7A—C3A—C4—C5	0.8 (3)	C7A—C7—C6—C9	−177.88 (19)
C3—C3A—C4—C5	−178.2 (2)	C8—C7—C6—C9	2.2 (3)
N2—O1—C7A—C3A	0.2 (2)	C7A—C3A—C3—N2	−0.4 (2)
N2—O1—C7A—C7	−178.95 (17)	C4—C3A—C3—N2	178.7 (2)
C4—C3A—C7A—O1	−179.26 (17)	C7A—C3A—C3—C10	179.5 (2)
C3—C3A—C7A—O1	0.1 (2)	C4—C3A—C3—C10	−1.4 (4)
C4—C3A—C7A—C7	−0.1 (3)	C3A—C4—C5—C6	−0.2 (3)
C3—C3A—C7A—C7	179.25 (18)	C7—C6—C5—C4	−1.2 (3)
C6—C7—C7A—O1	177.83 (18)	C9—C6—C5—C4	178.5 (2)
C8—C7—C7A—O1	−2.3 (3)	C3A—C3—N2—O1	0.5 (2)
C6—C7—C7A—C3A	−1.2 (3)	C10—C3—N2—O1	−179.36 (17)
C8—C7—C7A—C3A	178.7 (2)	C7A—O1—N2—C3	−0.5 (2)
C7A—C7—C6—C5	1.8 (3)	N2—C3—C10—Cl	−121.31 (19)
C8—C7—C6—C5	−178.1 (2)	C3A—C3—C10—Cl	58.8 (3)

### Hydrogen-bond geometry (Å, º)

**Table:**

*D*—H···*A*	*D*—H	H···*A*	*D*···*A*	*D*—H···*A*
C10—H10*B*···N2^i^	0.97	2.55	3.479 (3)	160

Symmetry code: (i) −*x*+1/2, −*y*+1/2, −*z*.
